# Protein Susceptibility to Peroxidation by 4-Hydroxynonenal in Hereditary Hemochromatosis

**DOI:** 10.3390/ijms24032922

**Published:** 2023-02-02

**Authors:** Sandra Sánchez-Jaut, Susana Pérez-Benavente, Paloma Abad, Darío Méndez-Cuadro, Antonio Puyet, Amalia Diez, Gonzalo Galicia-Poblet, Elena Gómez-Domínguez, María J. Moran-Jiménez, José M. Bautista, Isabel G. Azcárate

**Affiliations:** 1 Department of Biochemistry and Molecular Biology, Complutense University of Madrid, 28040 Madrid, Spain; 2 Research Institute Hospital 12 de Octubre, 28041 Madrid, Spain; 3 Analytical Chemistry and Biomedicine Group, School of Exact and Natural Sciences, San Pablo Campus, University of Cartagena, Cartagena 130014, Colombia; 4 Pediatric Digestive Service, Guadalajara University Hospital, 19002 Guadalajara, Spain; 5 Department of Medical Specialties and Public Health, Rey Juan Carlos University, 28922 Madrid, Spain

**Keywords:** hemochromatosis, oxidative stress, lipid peroxidation, 4-hydroxynonenal (HNE), Hfe^−/−^ mouse, erythrocyte membrane proteins, protein modification

## Abstract

Iron overload caused by hereditary hemochromatosis (HH) increases free reactive oxygen species that, in turn, induce lipid peroxidation. Its 4-hydroxynonenal (HNE) by-product is a well-established marker of lipid peroxidation since it reacts with accessible proteins with deleterious consequences. Indeed, elevated levels of HNE are often detected in a wide variety of human diseases related to oxidative stress. Here, we evaluated HNE-modified proteins in the membrane of erythrocytes from HH patients and in organs of Hfe^−/−^ male and female mice, a mouse model of HH. For this purpose, we used one- and two-dimensional gel electrophoresis, immunoblotting and MALDI-TOF/TOF analysis. We identified cytoskeletal membrane proteins and membrane receptors of erythrocytes bound to HNE exclusively in HH patients. Furthermore, kidney and brain of Hfe^−/−^ mice contained more HNE-adducted protein than healthy controls. Our results identified main HNE-modified proteins suggesting that HH favours preferred protein targets for oxidation by HNE.

## 1. Introduction

Iron overload in hereditary hemochromatosis (HH) causes tissue iron deposits that can lead to liver cirrhosis, chondrocalcinosis, arthritis, diabetes, hypogonadism, hepatocarcinoma or cardiomyopathy. HH results from molecular lesion at different genes. Recessive in-frame mutation C282Y in the homeostatic iron regulator gene (*HFE)* is a most prevalent polymorphic defect in the Caucasian populations. Some other rare forms involve genes encoding hemojuvelin (*HJV*) [1], hepcidin (*HAMP*) [2], transferrin receptor 2 (*TFR2*) [3] and ferroportin (*SLC40A1*) [4].

Iron toxicity is triggered by the accelerated generation of highly reactive hydroxyl radical (-OH) in the Fenton and Haber–Weiss reactions [5], causing oxidative damage to cells. Several studies have shown that oxidative stress plays an important role in the development of HH pathology [6,7,8,9,10,11,12], including ferroptosis, described as cell death elicited by lipid peroxidation under the catalysis of iron ions [13]. Increased circulating iron results in lipid peroxidation production above normal with increased protein oxidation [14,15,16]. Lipid peroxidation as a free radical chain reaction is induced by reactive oxygen species and produces a wide variety of oxidation products, among which HNE is the most toxic [5]. HNE reacts with several cellular components, such as DNA, proteins and other accessible molecules. High HNE concentrations cause cell cycle and differentiation arrest, concluding in cell death [17,18]. Although protein modification by HNE take place mainly by Michael addition to form covalent adducts with cysteine, lysine and histidine, alternatively, HNE can also form Schiff bases with amino groups [19]. Both types of HNE adducts contribute to protein cross-linking and carbonyl stress [5], resulting in altered protein function [20,21] and induction of antigenicity [22]. The consequence of protein oxidation and aggregate formation is eventually the disruption of cellular homeostasis and thus of the capacity for effective adaptive metabolic response.

Detection of protein modifications by HNE is used as a marker of oxidative stress [23]. A variety of diseases including chronic liver diseases [24], Alzheimer [25,26], Parkinson [27], cardiovascular damage [28], autism [29], diabetes [30] and infections [31] have been shown to increase levels of HNE-modified proteins, suggesting a role for their pathogenesis. Preliminary evidence of increased lipid peroxidation by iron overload has been shown in animal models of HH in cardiomyocytes and lung [32,33]. Furthermore, the direct relationship between toxicity by high level of lipid peroxidation with concurrent elevated iron levels in Parkinson’s disease [34,35], endothelial dysfunction and atherosclerosis [36,37,38], coronary heart disease [39] and intervertebral disc degeneration [40] has been observed.

During circulation, the erythrocyte membrane is continuously exposed to lipid peroxidation [41]. When oxidative stress occurs, oxidised proteins may be degraded by the erythrocyte 20S proteasome system. However HNE can inhibit proteasome activity through its binding to the enzymatic protein complex [42]. HNE binding to erythrocytes is able to cause their death [43,44]. Indeed, erythrocytes from HH patients carrying HFE mutations show eryptosis, a programmed erythrocyte death similar to apoptosis [44]. In addition, changes in the lipid profile of erythrocyte membranes due to increased lipid peroxidation have been observed in patients with HH [45]. This could explain the decreased cellular deformability, increased sensitivity to mechanical stress and the formation of dense fibrin deposits in erythrocytes of individuals with uncomplicated HH [46,47]. 

Two different mice models of HH have shown lipid peroxidative toxicity in organs, including liver, due to high F2-isoprostane [48] and HNE [32] levels. Moreover, iron accumulation in a model of HH type 4 promotes elevated lipid peroxidation in lung [33].

The aim of the present study was to determine the existence of potentially increased protein modification by HNE in the pathophysiological oxidative environment of HH disease. HNE is a common product of lipid peroxidation whose reactivity would contribute to protein dysfunction and thus to tissue damage and disease progression. The identification of biomarkers of oxidative status in the disease may contribute to earlier disease management. For that, we performed analyses of HNE-modified proteins from the erythrocyte membrane of HH patients and from Hfe^−/−^ mice organs to identify the most susceptible proteins of HNE-oxidative dysfunction. A high diversity of HNE-modified proteins was recognised in erythrocytes of HH patients. Moreover, kidney and brain from Hfe^−/−^ mice accumulated more HNE-adducted protein than the controls. These results are consistent with a higher oxidative status of HH and pointed out routes of dysfunction from the identified HNE targets.

## 2. Results

### 2.1. Identification of HNE-Modified Proteins in Erythrocyte Membrane of HH Patients

**Figure 1:**
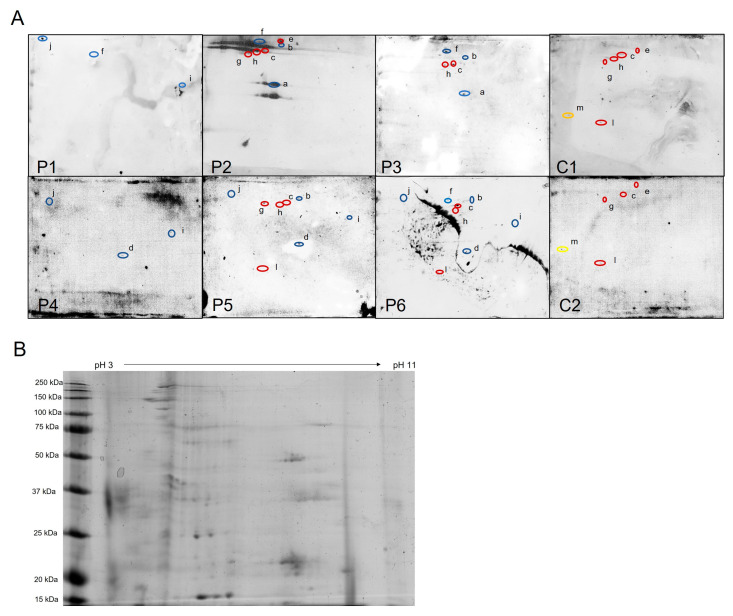
Identification of HNE-modified proteins from erythrocyte membrane of HH patients. (**A**) Western blots of HH patients (P1–P6) and healthy controls (C1 and C2). Protein spots identified by MALDI-TOF-TOF are designated with “a–m” letters and encircled with colours: red are spots present in patients and controls; blue spots are present only in HH patients; and yellow spots are only in controls. (**B**) Representative two-dimensional Coomassie brilliant blue-stained gel with total erythrocyte membrane proteins (Control sample 1). 4-hydroxynonenal (HNE), Hereditary hemochromatosis (HH).

Potential oxidative damage to erythrocyte membrane proteins in human HH was analysed by 2D immunoblotting to detect HNE oxidative adducts of isolated membrane proteins (Figure 1A). Spots of interest were excised and digested with trypsin from the stained spots at the Coomassie blue-stained duplicate gels (Figure 1B and Supplementary Figure S1) and analysed by MALDI-TOF/TOF to generate a peptide mass fingerprint.

Different HNE-bound protein spots were identified on erythrocytes samples from eight HH patients and healthy control people (Figure 1A). While five protein spots were common in both patients and controls (c, e, h, g, l), six protein spots were observed exclusively in the HH patients (a, b, d, f, I, j) and only one was present uniquely in the healthy controls (m). Patients numbered 1 to 4 were adults aged 41 to 50 years (see Section 4), while those numbered 5 and 6 were children aged 13 and 10 years.

Table 1 summarises the sample distribution of the nine identified proteins. The seven patient-specific proteins were guanine nucleotide-binding protein g(i)/g(s)/g(t) subunit beta-1, actin 1, actin 2, spectrin alpha chain, ankyrin 1, CD55 and CD44. The only two proteins found in both groups were spectrin beta chain and band 3 anion transport protein. No unique protein was found in the healthy controls.

Briefly, the proteins only identified in sample patients correspond to the following cell functions: guanine nucleotide-binding protein G(I)/G(S)/G(T) subunit beta-1 (P62873) is a modulator in transmembrane signalling systems required for the GTPase activity, for replacement of GDP by GTP and for G protein-effector interaction; cytoskeletal proteins actin cytoplasmic 1 and actin cytoplasmic 2 (beta actin, P60709 and gamma actin, P63261) contribute to cell motility and various biological processes such as sensing environmental forces, vesicular transport, moving over surfaces and cell division; ankyrin 1 (fragment H0YBS0; P16157) attaches integral membrane proteins to cytoskeletal elements; spectrin α chain (P02549) is the major constituent of the cytoskeletal network underlying the erythrocyte plasma membrane; CD55 (H3BLV0) regulates the complement system preventing the formation of the membrane attack complex; CD44 antigen (H0YD13) is a transmembrane glycoprotein involved in cell–cell communication, cellular adhesion and migration. Spectrin beta chain (H0YJE6, P11277) was identified in both HH patients and healthy individuals, which could suggest a high susceptibility of this protein to modification by HNE. In addition, band 3 anion transporter protein (P02730) was also found to be modified by HNE in both control and patient samples and being an important integral erythrocyte membrane glycoprotein could also act cooperatively with the cytoskeleton.

It should be noted that some proteins (spectrin beta chain, band 3 anion transport protein and ankyrin 1) were identified at more than one spot, suggesting post-translational modification or physiological proteolysis that results in molecular weight and/or charge of the corresponding protein.

### 2.2. Hfe^−/−^ Mice: A Model to Explore HH Tissue Damage

**Figure 2:**
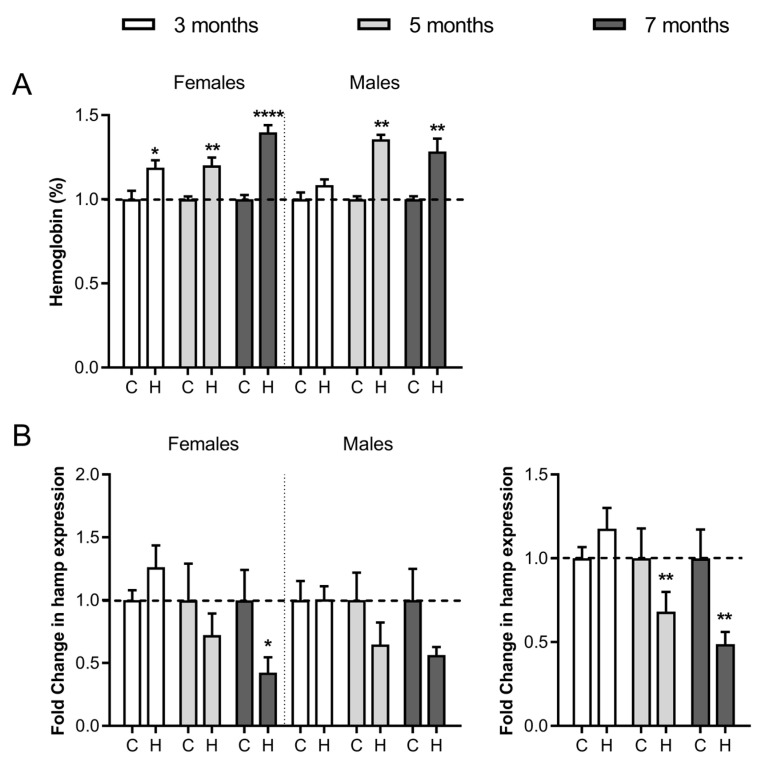
Haemoglobin levels and mRNA hepcidin expression in Hfe^−/−^ mice. (**A**) Normalised haemoglobin concentration in peripheral blood. (**B**) mRNA expression of hepatic hepcidin at 3, 5 and 7 months in Hfe^−/−^ (H) and wild type (C) female and male mice. *Hamp* (hepcidin gene) mRNA expression is also shown without gender separation (bottom right). The data for each Hfe^−/−^ mouse were normalised to controls of the same age and sex (indicated by dotted lines). Representative graphs show mean ± SEM from 2 independent experiments (each experiment *n* = 6 per bar/sex group). Statistical significance is indicated as **** *p* < 0.0001, ** *p* < 0.01 and * *p* < 0.05 in comparison to age- and sex-matched controls.

The previously described Hfe^−/−^ mouse model of HH [49,50,51] was followed at three time points (3, 5 and 7 months of age) and in both sexes to discriminate potential damage accumulated with age and in gender susceptibility. Haemoglobin concentration in peripheral blood was significantly higher in Hfe^−/−^ mice than in wild-type mice in both sexes at all ages (Figure 2A). Baseline haemoglobin values (mean ± standard deviation) for the control females were 12.98 ± 0.34 (g/dL) and for males 12.65 ± 0.31 (g/dL). Liver expression of *hamp1* mRNA, encoding peptide hepcidin-1 that limits intestinal iron absorption and iron recycling by macrophages to maintain iron homeostasis [52], was significatively reduced in Hfe^−/−^ mice at the 5- and 7-month age when considering both sexes (Figure 2B).

### 2.3. HNE Modifications in Kidney, Brain, Heart and Liver Proteins

**Figure 3:**
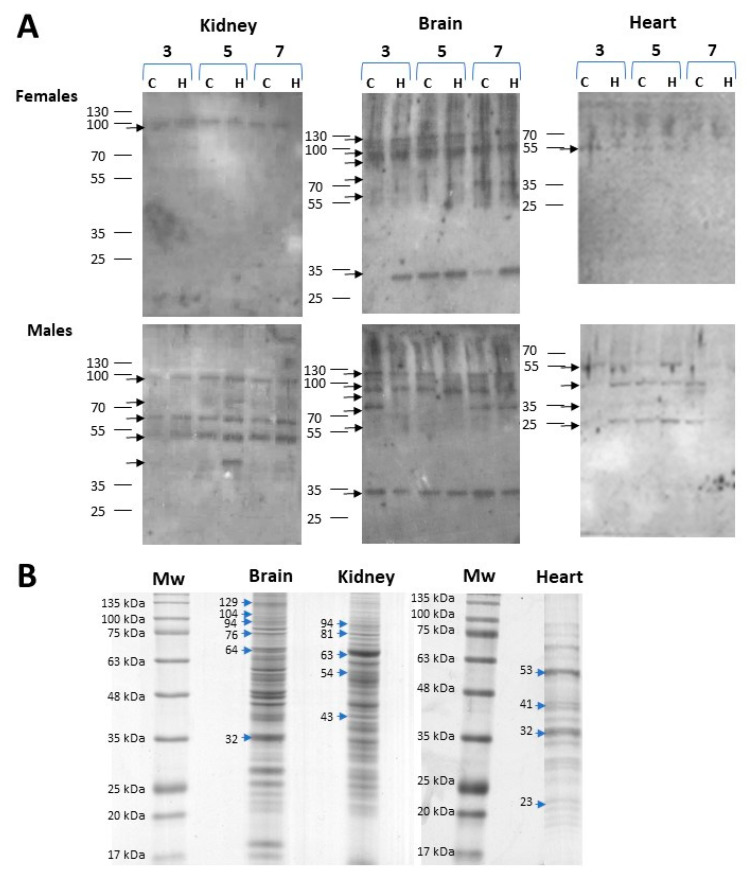
HNE-modified proteins in organs from female and male Hfe^−/−^ mice. Homogenates from each organ were separately resolved on 12% polyacrylamide gels. HNE (4-hydroxynonenal) modified proteins were detected by Western blot using HNE specific antibodies. (**A**) Western blot analysis of HNE adducts in kidney, brain, and heart proteins from female and male control (C) and Hfe^−/−^ (H) mice at 3, 5 and 7 months of age. Images are representative of *n* = 3 per group. (**B**) Coomassie brilliant blue-stained gels where HNE-immunoreactive bands protein bands were excised. Arrows indicate the protein bands that were excised from the gel and the molecular weight are expressed in KDa on the left side of the arrow.

The extent of protein carbonylation mediated by HNE in the kidney, brain, heart and liver of male and female Hfe^−/−^ mice at 3, 5 and 7 months of age was assessed by Western blotting using anti-HNE antibodies. More differences were found in the protein carbonylation patterns of each gender than between the Hfe^−/−^ and control mice. A pattern of five bands of modified kidney proteins at 94, 81, 63, 54 and 43 kDa was distinguished in males but only a 94 kDa band in females (Figure 3A). The greatest HNE immunoreactivity with high number of protein bands and strong signal was observed in the brain. Both female and male mice showed six bands at 129, 104, 94, 76, 64 and 32 kDa. Furthermore, while four HNE-immunoreactivity bands were found in male heart at 53, 41, 32 and 23 kDa, such signals were barely visible in females. On the other hand, HNE signals in liver were weak and almost imperceptible in both sexes, hence, this organ was not considered in protein identification studies. Immunoblots of biological replicates are shown in Supplementary Figures S2–S7. To further identify HNE-modified proteins in the kidney, brain and heart, the corresponding bands were excised from the Coomassie brilliant blue-stained parallel gel, digested, immuno-enriched and analysed by MALDI TOF/TOF.

The corresponding HNE-immunoreactive protein bands from each organ, as shown in Figure 3B, were excised from the gel for MALDI TOF/TOF analysis and most were identified (Table 2). In the kidney, although five HNE-modified proteins were identified, only the 94 kDa band was immunoreactive in females (Figure 3A). Briefly, at band 1, mitochondrial sarcosine dehydrogenase (EC 1.5.8.3.) converts sarcosine (N-methylglycine) to glycine; at band 2, mitochondrial aconitase hydratase (EC 4.2.1.3) interconverts citrate and isocitrate; at band 3, serum albumin regulates blood osmotic pressure and is a transporter of cations, fatty acids, hormones, bilirubin and drugs; at band 4, two proteins were identified, methanethiol oxidase (EC 1.8.3.4), a modulator of cellular redox homeostasis, and mitochondrial methylmalonate-semialdehyde dehydrogenase (EC 1.2.1.27) was involved in valine and pyrimidine metabolism; and at band 5 NADP-alcohol dehydrogenase (EC 1.1.1.2) catalysing the reduction of a wide variety of carbonyl-containing compounds to their corresponding alcohols (Table 2).

In the brain, the six HNE-immunoreactive bands were equally detected in male and female mice (Figure 3A). Bands 1 and 4 were both identified as Na^+^/K^+^-exchanging ATPase subunit alpha-3 which, as a complete enzyme (EC 7.2.2.13), catalyses the hydrolysis of ATP coupled with the exchange of sodium and potassium ions across the plasma membrane (Table 2). Band 3, mitochondrial aconitase hydratase (EC:4.2.1.3), catalyses the conversion of citrate to isocitrate in the tricarboxylic acid (TCA) cycle. In band 5, two proteins were identified: heat shock cognate 71 kDa protein with a key role in protein quality control system and serum albumin, which was also found in kidney as band 3. In band 6 was identified mitochondrial malate dehydrogenase (EC:1.1.1.37) which, as the final step in the TCA cycle, reversibly catalyses the NAD/NADH-dependent oxidation of malate to oxaloacetate.

In the heart, a higher number of bands detected by anti-HNE antibody was found in male than in female mice (Figure 3A). Within band 1, the alpha and beta subunits of mitochondrial membrane ATP synthase (F1F0 ATP synthase or Complex V: EC 7.1.2.2) were identified in males and females (Table 2). The rest of HNE-immunoreactive bands were only found in male tissue: band 2, mitochondrial creatine kinase-S-type (EC:2.7.3.2), catalyses the transfer of phosphate between ATP and various phosphogens (e.g., creatine phosphate) playing a central role in energy transduction; band 3, mitochondrial malate dehydrogenase (EC 1.1.1.37) which was also found in the brain as band 6; band 4, with two proteins identified: mitochondrial ATP synthase F(0) complex subunit B1 (F1F0 ATP synthase or Complex V: EC 7.1.2.2) and apolipoprotein A-I, this last participating in the reverse transport of cholesterol from tissues to the liver for excretion.

**Figure 4:**
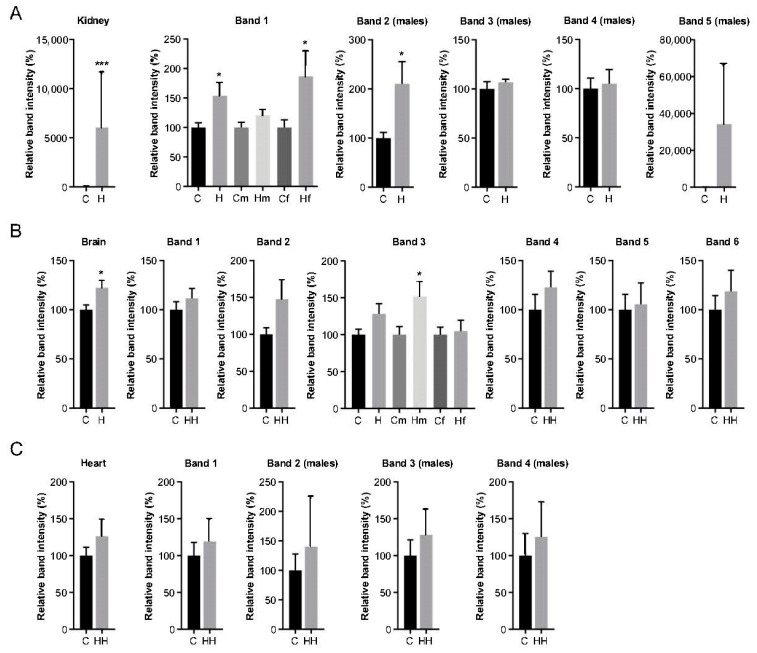
Densitometric analyses of 4-hydroxynonenal (HNE)-protein signals. Global and individual intensities of bands as shown in Figure 3A and identified in Table 2 from (**A**) kidney, (**B**) brain and (**C**) heart of control (C) and Hfe^−/−^ (H) mice. Analysis of male (m) and female (f) mice is shown separately only in cases of statistically significant results. When band analysis is shown for males only, it is indicated in brackets (males) at the top. Total Hfe^−/−^ mice or control *n* = 18; male/female Hfe^−/−^ or control mice *n* = 9. Values are expressed as relative band intensity (%) considering 100% the mean value of *n* = 18 control mice or *n* = 9 male/female control mice as indicated (mean ± SEM). Statistical comparisons were performed between groups using Student unpaired *t*-tests or Mann–Whitney U-tests. * *p* < 0.05, *** *p* ≤ 0.001.

Additionally, semi-quantitative analysis of anti-HNE reactivity signals of Hfe^−/−^ in comparison to control samples (ran in the same Western blot: Figure 3A and Supplementary Figures S2–S7) was performed by densitometry of those bands listed in Table 2. Since band intensity remained unchanged within age groups, data are shown grouped according by HH status although, when statistically significant, they are also compared by sex (Figure 4).

In the kidney, levels of total HNE-modified protein signal from Hfe^−/−^ mice were significantly higher (*p* = 0.001) than those found in healthy mice (Figure 4A, kidney). Of the five HNE-modified protein bands from the kidney, only mitochondrial sarcosine dehydrogenase was also present in females (Figure 3A). Its band intensity shown in Figure 4A (band 1) of all Hfe^−/−^ mice irrespective of gender (*p* = 0.0156) and in Hfe^−/−^ females (*p* = 0.04) was significantly higher than in the control mice. Similarly, HNE modification was greater in aconitase hydratase protein (Figure 4A band 2) from male Hfe^−/−^ mice (*p* = 0.022) than in the matched control mice. In the brain, the six bands observed in both sexes showed an overall increase in HNE-protein signal in Hfe^−/−^ mice than in the controls (Figure 4B brain). However, when treated individually, only mitochondrial aconitate hydratase of males showed a significant intensity increase than in the control mice (Figure 4B band 3). Finally, the HNE signal of the heart proteins showed no differences between Hfe^−/−^ and the control mice, either globally (Figure 4C heart) or in any of the individual bands.

### 2.4. Hepatic Expression of Iron-Related mRNA Genes in Hfe^−/−^ Mice

**Figure 5:**
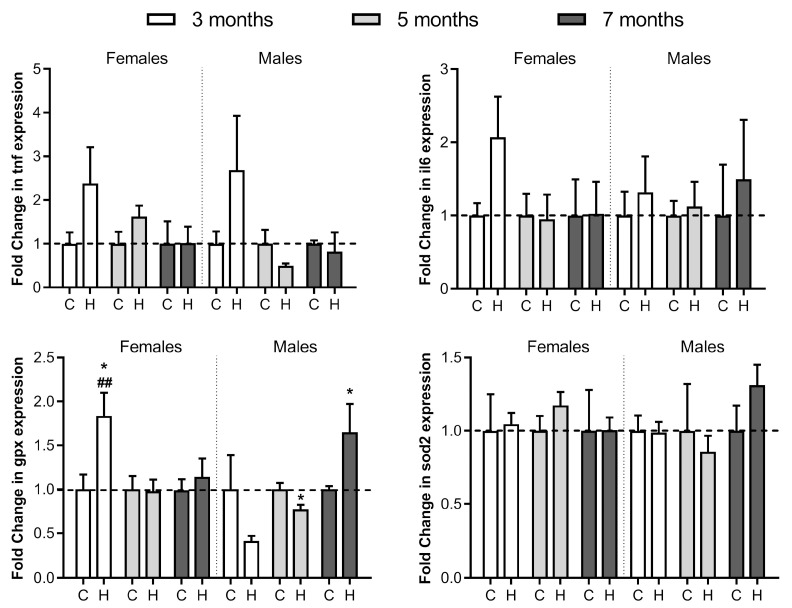
Comparative liver mRNA expression of *tnf*, *il6*, *gpx1* and *sod2*. Relative expression of tumour necrosis factor-α (*tnf*), interleukin 6 (*il6*), glutathione peroxidase-1 (*gpx1*) and superoxide dismutase 2 (*sod2*) in female and male 3-, 5- and 7-month-old Hfe^−/−^ mice (H) and healthy controls (C) was calculated by the 2^−ΔΔCT^ method. The results are expressed as arbitrary units normalised to β-actin to correct for mRNA quantity and integrity. The dotted lines represent normalized values of healthy controls. Data are shown as mean ± SEM, each with *n* = 6 per group, where * *p* ≤ 0.05 comparing to same-age controls, and ^##^ *p* < 0.01 compared to same age Hfe^−/−^ mice but different gender.

To understand liver dysfunction caused by HH in mice associated to the oxidative modifications observed, the expression of a group of four genes related to the disease was also analysed at 3, 5 and 7 months of age (Figure 5). Hepatic gene expression of two cytokines, tumour necrosis factor-α (*tnf*) acting as hepcidin mRNA inhibitor [53] and interleukin 6 (*il6*) acting as hepcidin mRNA inducer [54] showed no significant changes. Glutathione peroxidase-1 (*gpx1*) and superoxide dismutase 2 (*sod2*) encoding two oxidative stress response enzymes showed some changes. Thus, *gpx1* at 3-month-old female Hfe^−/−^ mice showed a significant enhanced *gpx1* expression compared to the control and Hfe^−/−^ males of the same age. Hfe^−/−^ males showed a lower but significative *gpx1* expression at 5 months of age, but notably increased at 7 months of age. In the case of *sod2*, no expression differences were observed between any group.

## 3. Discussion

Our study shows a total of seven membrane proteins modified by HNE exclusively in HH patients, whereas no HNE target protein was identified singularly in healthy controls, suggesting a more oxidative circulating environment in HH. Furthermore, since HNE easily crosses membranes and protein-HNE adducts are detectable in all cellular compartments [55], the increased carbonylation observed in HH patients may probably be extensible to the interior of the red cell.

Two of the proteins modified in HH patients in this study were guanine nucleotide-binding protein g(i)/g(s)/g(t) subunit beta-1, and actin, both described as carriers of HNE binding sites [56,57]. Actin-HNE adducts in red cell membrane have also been detected in autism, suggesting a link between erythrocyte shape abnormalities, membrane oxidative stress damage and actin alteration [29]. Ankyrin that was observed as HNE modified in HH patients has been found carbonylated in renal disease, G6PD deficiency, sickle cell trait or malaria infection [58,59,60], suggesting a highly susceptible cytoskeleton protein in oxidative environments. Spectrins, also components of the membrane skeleton bound to ankyrin, band 4.1 and actin, and responsible for erythrocyte shape and membrane lipid asymmetry [61,62], were found to be modified in the HH patients and the β-chain was also found in the healthy patients, consistent with being a main target of HNE adduction in intact exposed erythrocytes [63]. Our results suggest a link between oxidative damage of erythrocyte membrane proteins and erythrocyte shape abnormalities [46,47], described in HH as caused by structural defects in the membrane skeleton [64] and in HNE-treated erythrocytes [65]. On the other hand, the biological significance of the HNE modifications found in CD55 and CD44 antigens that were unique to erythrocytes from HH patients may be related to the reported increased intravascular haemolysis associated with an increased oxidative state that reduces CD55 expression [66]. A limitation of our study is the small number of human samples available. It would be desirable to increase the biological samples per group to associate the potential presence and amount of HNE-protein adducts with the pathology of the disease.

Band 3 anion transporter protein detected at different spots on 2D gels in both HH patients and healthy controls can be explained by the fragmentation of both of its domains [67] as described during erythrocyte senescence [68]. In fact, both fragment sizes are distinguished in fresh extracts from erythrocyte membrane proteins [68]. Alternatively, as a glycoprotein, the multiple spots of band 3 anion transporter could be due to a heterogeneous carbohydrate glycosylation shown by different mobilities by isoelectric focusing [69]. Moreover, since spectrin was found in three spots (f, g and h), with two of them (g and h) containing only its beta chain while the highest molecular weight spot (f) contained its alpha chain together with an ankyrin 1 fragment, it can be hypothesised that these different associated cytoskeleton spots are due to the retention of part of their original bonds even after electrophoresis [62].

In HH patients, the symptoms usually become apparent after 40–60 years of age [70] and biochemical markers are progressively altered with age [71]. To study the increased toxicity induced by the lipid peroxidative product HNE in organs, Hfe^−/−^ mice was chosen as a model of progressive increase in hepatic and plasma iron concentrations, ferritin alteration and transferrin saturation over the course of months [72,73,74] as HH is a disease in which iron accumulates in different tissues, increasing oxidative stress. However increased oxidation was not observed with an age difference of 4 months in C57BL/6J mice, which as an aging model [75,76,77,78,79] may not be sufficient to show a cumulative effect as the few studies analysing the age-related formation of 4-HNE adducts [80] or ROS [81] in wild-type C57BL/6J mouse tissues show some difference only at 12 months of age. Therefore, our results in the first half of the mouse life span seem to correspond to the late onset of iron accumulation and oxidative products in humans and, therefore, this mouse model may allow us to observe those early changes that can later lead to accumulated tissue damage. Thus, in our study, increased oxidative stress damage in Hfe^−/−^ mice was shown early by the high presence of HNE-modified proteins in the kidney and brain in comparison to the controls. This is consistent with the observed cause of kidney injury by circulating iron [82,83] and the increased oxidative stress environment observed in the brain of mice carrying an HFE mutation [84]. In this regard, human renal impairment has been described in some HH patients [85,86], while patients carrying the HFE C282Y mutation in homozygosis develop marked iron deposition in dementia-relevant brain areas [87].

HNE-immunoblot analysis of kidney, brain, liver and heart proteins showed that, among all proteins, mitochondrial sarcosine dehydrogenase in Hfe^−/−^ female kidney (band 1) and mitochondrial aconitase hydratase in kidney (band 2) and brain (band 3) of Hfe^−/−^ male had the highest and most distinct signals relative to those corresponding to wild-type mice. Sarcosine dehydrogenase produces free formaldehyde in the conversion of sarcosine to glycine and its HNE adducts have been already described [88]. It has been described that aconitase, a key enzyme of the Krebs cycle, undergoes reversible inactivation by ROS [89,90], being a known biomarker of mitochondrial oxidative stress [91], since in addition to being an essential organelle for ATP generation, it is also a major producer of ROS [92]. Iron-driven oxidation requires direct interaction with cellular reducing and oxidizing equivalents such as the enriched mitochondrial with superoxide and hydrogen peroxide suppliers of electrons. Consequently, increased oxidation is likely to occur in iron and free electron overloaded microenvironments such as mitochondria, where its dysfunction would be mediated [93,94].

Beyond our objectives, we observed sex-specific differences in the number of HNE-adducted proteins in the kidney and heart. A higher level of oxidative stress injury induced by iron overload has been previously described in male mice compared to females in HJV knockout mice with high-iron diets [95], and in mice inoculated with iron-disodium salt [96]. The kidney proteins aconitase hydratase, albumin, methanethiol oxidase, mitochondrial methylmalonate-semialdehyde dehydrogenase and alcohol dehydrogenase were observed to be HNE modified only in male mice. Therefore, although it is not known whether it is more frequent in men, the observation of an acceleration of renal disease progression by elevated lipid peroxidation products such as HNE and malondialdehyde [83,97] is relevant. In the heart, mitochondrial enzymes creatine kinase- S-type and malate dehydrogenase, as well as apolipoprotein A-I were uniquely HNE modified in males. These early increased oxidative stress markers identified in male Hfe^−/−^ mice may differentially contribute to the mitochondrial dysfunction recognised as key player in cardiac diseases [98].

Our study is the first to describe the presence of the lipid peroxidation product HNE in erythrocytes from HH patients. In the present study, we have discovered HNE-modified proteins in the membrane of erythrocytes from HH patients associated with structural functions of the cytoskeleton and membrane receptors that have previously been linked to the maintenance of erythrocyte shape and appearance. In addition, we studied the presence of HNE in different tissues of Hfe^−/−^ mice and it was the kidney and brain that had more modified proteins than the healthy controls. Our study provides evidence for an increased presence of HNE-protein adducts in HH disease. Thus, the determination of certain HNE-bound erythrocyte proteins in the clinical follow-up of HH patients could be useful as a marker of oxidative toxicity and for monitoring disease progression. Moreover, even further investigation of the potential loss of activity of HNE-modified proteins that have been identified in mouse organs could contribute to the understanding of the pathogenesis of HH sequelae. Thus, it would be worthwhile to further study the presence of erythrocyte and tissue markers related to oxidative stress to assist with treatment and to more precisely monitor the pathophysiological status of patients.

## 4. Materials and Methods

### 4.1. Human Subjects and Animal Model

Blood samples were obtained from six HH patients (Table 3). Patients 4, 5 and 6 were family related, being father, daughter and son, respectively. All HH patients were diagnosed by genetic testing. Two healthy volunteers served as a healthy control group. This study was approved by the Ethical Review Board at Research Institute Hospital 12 de Octubre, Madrid (Spain) (ethical approval no. 14/400 03 06 2015) and was conducted according to the guidelines laid down by the Helsinki Declaration, with written informed consent obtained for each adult participant or, in the case of children, a parent or a guardian of the child participant provided written informed consent on their behalf or the child’s assent. 

A total male (*n* = 18) and female (*n* = 18) Hfe^−/−^ mice on a C57BL/6J background (strain B6 129P2-Hfetm1gfn ⁄J from The Jackson Laboratory, Bar Harbor, ME, USA) [50] and male (*n* = 18) and female (*n* = 18) Hfe^+/+^ mice of the same genetic background were used as the healthy control (*n* = 6 per group of sex, age and HH condition). Age groups were 3, 5 and 7 months old. Animals were bred under standard conditions and supplied with food and water ad libitum. A standard rodent chow was chosen with an iron content of 160 mg/kg (Altromin LASQCdiet Rod14-H, Soest, Germany). All the experiments with animals were approved by the Committee of Animal Experimentation of the Universidad Complutense de Madrid in agreement with National (R.D. 53/2013) (ethical approval no. O.H. (CEA)-UCM-15-2017) and European (2010/63/CE) legislation.

### 4.2. Hemoglobin Concentration

To determine the hemoglobin concentration, a drop of blood from each subject or animal was placed into the HemoCue Hb 301 analyzer (HemoCueAB, Angelholm, Sweden). 

### 4.3. Isolation of Human Erythrocyte Membrane Proteins

Erythrocyte membrane protein isolation followed previously published procedures [59,99]. Briefly, peripheral whole blood was stored for 5 days at 4 °C to allow maturation of reticulocytes to erythrocytes. Serum was discarded and RPMI (Catalog: #31800022, Thermo Fisher Scientific, Waltham, MA, USA), HEPES 25 mM (Catalog: #10204932, GE Healthcare Life Sciences, Chicago, IL, USA) (*v*/*v*) at pH 7.4 was added in a 1:1 ratio. Diluted blood cells were suspended in Lymphoprep (Catalog: #07811, Stemcell, Vancouver, BC, Canada) in a 1:1 ratio and centrifuged at 800× *g* for 20 min. White cell fraction were removed and erythrocytes were washed twice with RPMI with 2.5% HEPES (*v*/*v*) and 100 μM of 3,5-di-tert-4-butylhydroxytoluene (BHT; Catalog: #47168, Sigma, St Louis, MO, USA) to avoid oxidative modifications. Erythrocytes were washed in phosphate buffer and used to obtain membrane proteins. Hemolysate supernatants obtained at 9000× *g* during 20 min at 4 °C were discarded and washed and centrifugation was repeated (at least 5 times) until supernatants appeared colourless. 15 mL of 100 mM sodium carbonate (Na_2_CO_3_) pH 11.0 was added to the precipitate and passed 5 times through a 25G needle after which it was kept in gentle agitation for 30 min at 4 °C and centrifuged at 20,000× *g* at 4 °C for 20 min. Membrane ghosts were resuspended in modified RIPA buffer with 50 mM Tris 50 mM NaCl, 3% 3-[(3-Colamidopropyl)-dimethylammonium]-propanesulfonate (CHAPS) (*w*/*v*), 0.5% decanoyl-N-methylglucamide (MEGA 10) (*w*/*v*), 100 μM BHT and a protease inhibitor cocktail (Catalog: #4693159001, Roche, Basel, Switzerland) and shaken vigorously every 5 min for 1 h keeping it at 4 °C. Finally, it was centrifuged for 1 h at 7000× *g* at 4 °C, and the supernatant was collected. Protein concentration was determined using a modified Bradford method (Catalog: #5000201, Bio-Rad, Hercules, CA, USA).

### 4.4. Protein Extracts from Mouse Tissues

Liver, kidneys, brain and heart were immediately dissected upon animal sacrifice and stored in modified RIPA buffer at −80 °C. For extract preparation organs were thawed on ice and disintegrated. Then were shaken vigorously every 5 min for 1 h at 4 °C and centrifuged for 1 h at 7000× *g* and the soluble supernatant was collected for analyses.

### 4.5. Two-Dimensional Electrophoresis and Immunoblot of Human Erythrocyte Membrane Proteins

For identification of post-translational modifications of erythrocyte membrane proteins, 55 μg of membrane protein fractions obtained from each patient were separated by two-dimensional electrophoresis on 12% polyacrylamide (*w*/*v*) gels under denaturing conditions. Subsequently, the two-dimensionally separated proteins were transferred to nitrocellulose membranes that were incubated in PBS containing 5% skimmed milk powder (*w*/*v*) and 0.05% polysorbate 20 (Tween-20) (*v*/*v*), for 30 min at room temperature on an orbital shaker. Later, nitrocellulose membranes were incubated with anti-HNE polyclonal antibodies (gently provided by Dr. Dario Méndez [31]) 1:500 16 h at 4 °C, washed and incubated with the peroxidase-conjugated secondary antibodies 1:4000 (Catalog: #GENA9310-1ML, Amersham ECL Mouse IgG, HRPlinked F(ab’)_2_ fragment; GE Healthcare Life Sciences, Chicago, IL, USA) for 1 h. After washing, antigen-antibody interaction was detected by chemiluminescence using the Western Lightning ECL Pro substrate (Catalog: #NEL120E001EA, Perkin Elmer, Waltham, MA, USA) for 5 min. Light emission from the reaction was detected in the AGFA CP 1000 equipment (AGFA). PDQuest software (Bio-Rad, Hercules, CA, USA) was used to analyse protein spots. Parallel run 2D gels were stained with Colloidal Coomassie Blue Staining Kit (Catalog: #LC6025, Invitrogen, Waltham, MA, USA) to compare with the membranes and then to excise spots of interest.

### 4.6. One-Dimensional PAGE Electrophoresis and Immunoblot

Protein extracts from the different mouse tissues that were obtained were loaded in one-dimensional PAGE electrophoresis using precast denaturing 12% polyacrylamide (*w*/*v*) Mini-PROTEAN TGX™ Precast gels (Catalog: #4568043, Bio-Rad, Hercules, CA, USA). Samples (25 μg) were prepared in loading buffer containing 50 mM Tris (pH 6.8), 250 mM DTT, 2% SDS (*w*/*v*) and 0.25% bromophenol blue (Catalog: #1610404, BioRad, Hercules, CA, USA). Once proteins were separated, gels were transferred onto nitrocellulose membranes. Identification of HNE-modified proteins followed identical methodology using anti-HNE antibodies than that described in the previous section.

### 4.7. MALDI-TOF/TOF Analysis

Selected 1D or 2D gel spots either from human or mouse samples were manually excised and automatically digested using the Proteineer (Bruker Daltonics, Billerica, MA, USA). For MALDI-TOF/TOF analysis, samples were automatically acquired in an ABI 4800 MALDI-TOF/TOF mass spectrophotometer (Applied Biosystems, Waltham, MA, USA) as previously described [100]. The data obtained were subjected to search with the MASCOT version 2.3 algorithm with the constraints of decarboxyamidomethylation and species.

### 4.8. Analysis of mRNA Expression

Mice hepatocyte mRNA (*n* = 6 per group) was extracted to determine the expression of *hamp*, *tnf*, *il6*, *sod2* and *gpx*. The tissue was previously stored at −80 °C in sections of approximately 2–5 mm in 300 µL of RNAlater solution (Catalog: #AM7020, Thermo Fisher Scientific, Waltham, MA, USA). mRNA was extracted in duplicate using the GeneJET RNA purification kit (Catalog: #K0702, Thermo Fisher Scientific, Waltham, MA, USA) including DNAse I (AM1906 Thermo Fisher Scientific, Waltham, MA, USA) digestion according to the manufacturer’s instructions. Subsequently, the cDNA was synthesized using the High-Capacity cDNA Reverse Transcription Kit (Catalog: #4368814, Applied Biosystems, Waltham, MA, USA) for Real-Time Quantitative Reverse Transcription PCR (qRT-PCR) in an ABI 7000 Sequence Detection System (Applied Biosystems, Waltham, MA, USA). PCR reactions were performed for commercial mixtures of primers and specific probes corresponding to the sequences of the *hamp*, *tnf*, *il6*, *sod2*, *gpx* and β-*actin* gene, which was used as constitutive expression (Catalog: #4331182, Applied Biosystems assays Table 4). All PCR reactions were set with Maxima Probe/ROX qPCR Master Mix (2×) (#K0231, Thermo Fisher Scientific, Waltham, MA, USA). PCR reactions included an uracil DNA glycosylase pre-treatment of 2 min at 50 °C, an initial incubation of 10 min at 95 °C for polymerase activation, followed by 40 cycles (melting 15 s at 95 °C, annealing and extension 1 min at 60 °C). Relative changes in gene expression were calculated using the comparative 2^−ΔΔCT^ method. Water was used as a negative control.

### 4.9. Statistical Analysis

Statistical analysis of quantitative variables was performed using the t Student parametric test or Mann–Whitney nonparametric test to find significant differences between groups. The Shapiro–Wilk test was used to calculate the normal distribution. Bartlett’s test was used for homogeneity of the variance. All statistical analyses were performed using GraphPad Prism 9 (GraphPad Software Inc., San Diego, CA, USA). A *p* < 0.05 was considered significant.

## Figures and Tables

**Table 1:** HNE-modified membrane proteins from erythrocytes of HH patients.

Protein Name	UniProt ID	Mascot Score	Spot	Patients	Nº Samples
▪ **HH patients (blue)**					
Guanine nucleotide-binding protein g(i)/g(s)/g(t) subunit beta-1 *	P62873	108	a	P2, P3	2
Unidentified			b	P2, P3, P5, P6	4
Actin, cytoplasmic 1 * and Actin, cytoplasmic 2 * *(a)*	P63261 P60709	117 117	d	P4, P5, P6	3
Ankyrin 1 (Fragment) Spectrin alpha chain, erythrocytic 1 * Spectrin beta chain (fragment)	H0YBS0 P02549 H0YJE6	271 121 113	f	P1, P2, P3, P6	4
Ankyrin 1 *	P16157	71	i	P1, P4, P5, P6	4
CD55 (Fragment) * CD44 Antigen *	H3BLV0 H0YD13	103 80	j	P1, P4, P5, P6	4
▪ **HH patients and healthy individuals (red)**					
Unidentified			c	P2, P3, P5, P6, C1, C2	6
Band 3 anion transport protein	P02730	258	e	P2, C1, C2	3
Spectrin beta chain, erythrocytic Spectrin beta chain, erythrocytic	P11277 H0YJE6	306 356	h	P2, P3, P5, P6, C1	5
Spectrin beta chain, erythrocytic (fragment)	H0YJE6	356	g	P2, P5, C1, C2	4
Band 3 anion transport protein	P02730	248	l	P5, P6, C1, C2	4
▪ **Healthy individuals (yellow)D**					
Band 3 anion transport protein	P02730	617	m	C1, C2	2

* Only found in HH patients. *(a)* These two accession numbers from UniProt were scored simultaneously since have an identity of 99% (371 in 375 residues, both of the same length) and as such were undistinguishable in the MADI-TOF/TOF spectra obtained for most of the fragments to discriminate both.

**Table 2:** Identification of 4-HNE-modified proteins in Hfe^−/−^ mice organs.

Band Nº	Protein Name	UniProt ID	MW (KDa)	Gender
**Kidney**				
1	Sarcosine dehydrogenase, mitocondrial *	Q99LB7	94	F, M
2	Aconitase hydratase, mitocondrial *	Q99KI0	81	M
3	Albumin	P07724	63	M
4	Methanethiol oxidase Methylmalonate-semialdehyde dehydrogenase (acylating), mitochondrial	P17563 Q9EQ20	54	M
5	Alcohol dehydrogenase (NADP+)	Q9JII6	43	M
**Brain**				
1	Sodium/potassium-transporting ATPase subunit alpha-3	Q6PIC6	130	F, M
2	UNIDENTIFIED		120	F, M
3	Aconitate hydratase, mitocondrial *	Q99KI0	110	F, M
4	Sodium/potassium-transporting ATPase subunit alpha-3	Q6PIC6	75	F, M
5	Heat shock cognate 71 kDa protein Albumin	P63017 P07724	64	F, M
6	Malate dehydrogenase, mitochondrial	P08249	32	F, M
**Heart**				
1	ATP synthase subunit alpha, mitochondrial ATP synthase subunit beta, mitochondrial	Q03265 P56480	53	F, M
2	Creatine kinase- S-type mitochondrial	Q6P8J7	41	M
3	Malate dehydrogenase mitochondrial	P08249	32	M
4	ATP synthase F(0) complex subunit B1 mitochondrial Apolipoprotein A-I	Q9CQQ7 Q00623	23	M

F, female; M, male. * Differences between Hfe^−/−^ and control mice were detected by densitometric quantification of HNE-Western blot bands.

**Table 3:** Patient and control data.

	HH	Sex	Age (Years)	Phlebotomy
**Patients**				
1	HH1	Male	50	Y
2	Unknown	Female	53	Y
3	HH1	Male	50	Y
4	HH4	Male	41	N
5	HH4	Female	13	Y
6	HH4	Male	10	Y
**Control**				
1	Healthy	Male	57	N
2	Healthy	Female	32	N

**Table 4:** qRT-PCR primer and probe data.

Assay	Gene	Gene Accession Number	Amplicon Size
Mm04231240_s1	*Hamp*	84506	86
Mm00443258_m1	*Tnf*	21926	81
Mm00446190_m1	*Il6*	16193	78
Mm01313000_m1	*Sod2*	20656	67
Mm00656767_g1	*Gpx1*	14775	134
Mm01205647_g1	*Actb*	11461	72

## Data Availability

Data are contained in the article.

## Supplementary Materials

The following supporting information can be downloaded at: https://www.mdpi.com/article/10.3390/ijms24032922/s1.
