# Supplementary material for: Protein Susceptibility to Peroxidation by 4-Hydroxynonenal in Hereditary Hemochromatosis
Source: Int J Mol Sci. 2023 Feb 2;24(3):2922. doi: 10.3390/ijms24032922 (PMC9917916; doi:10.3390/ijms24032922)
Supplement: Supplementary file 1 [file ijms-24-02922-s001.zip › ijms-2174383-supplementary.pdf]

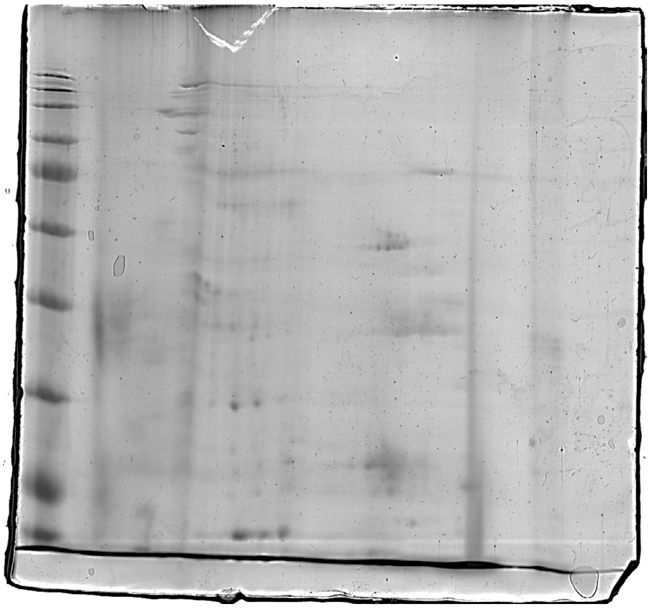

Supplementary Figure S1. Original two-dimensional Coomassie brilliant blue-stained gel with total erythrocyte membrane proteins from human control 1.

Kidney

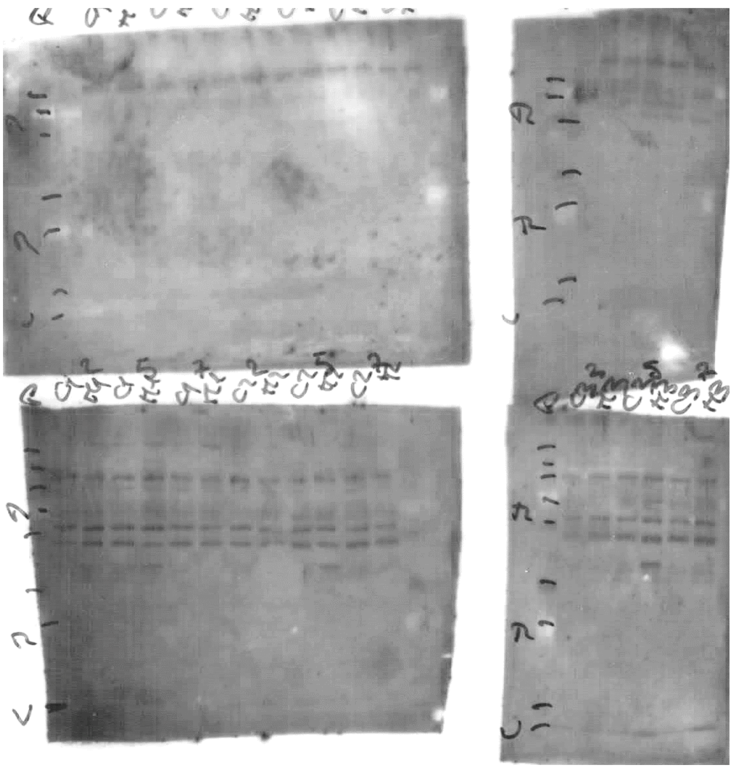

Supplementary Figure S2. Original uncropped Western blot analysis of HNE-modified proteins in kidney. Blots are labelled in supplementary figure 3. Parts of blots are separately presented in Figure 3A.

Kidney

A

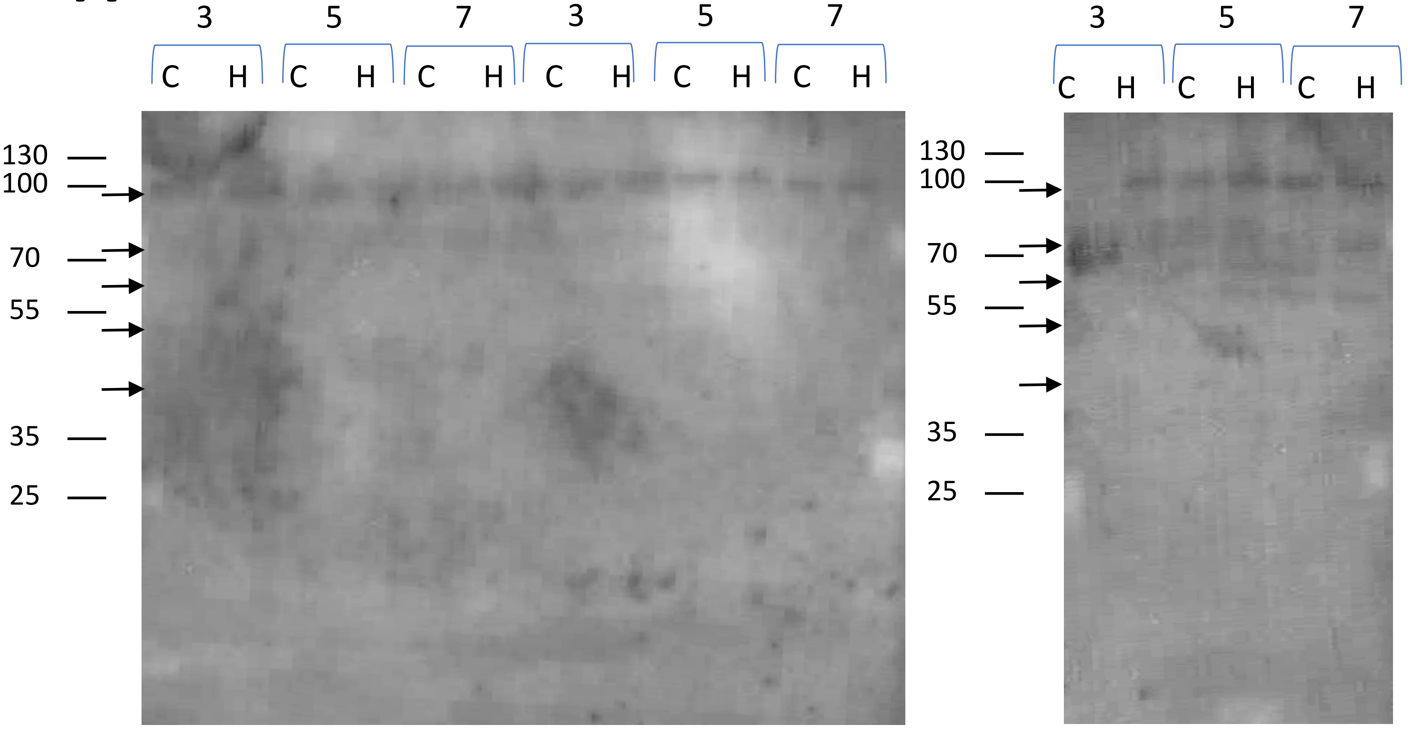

B

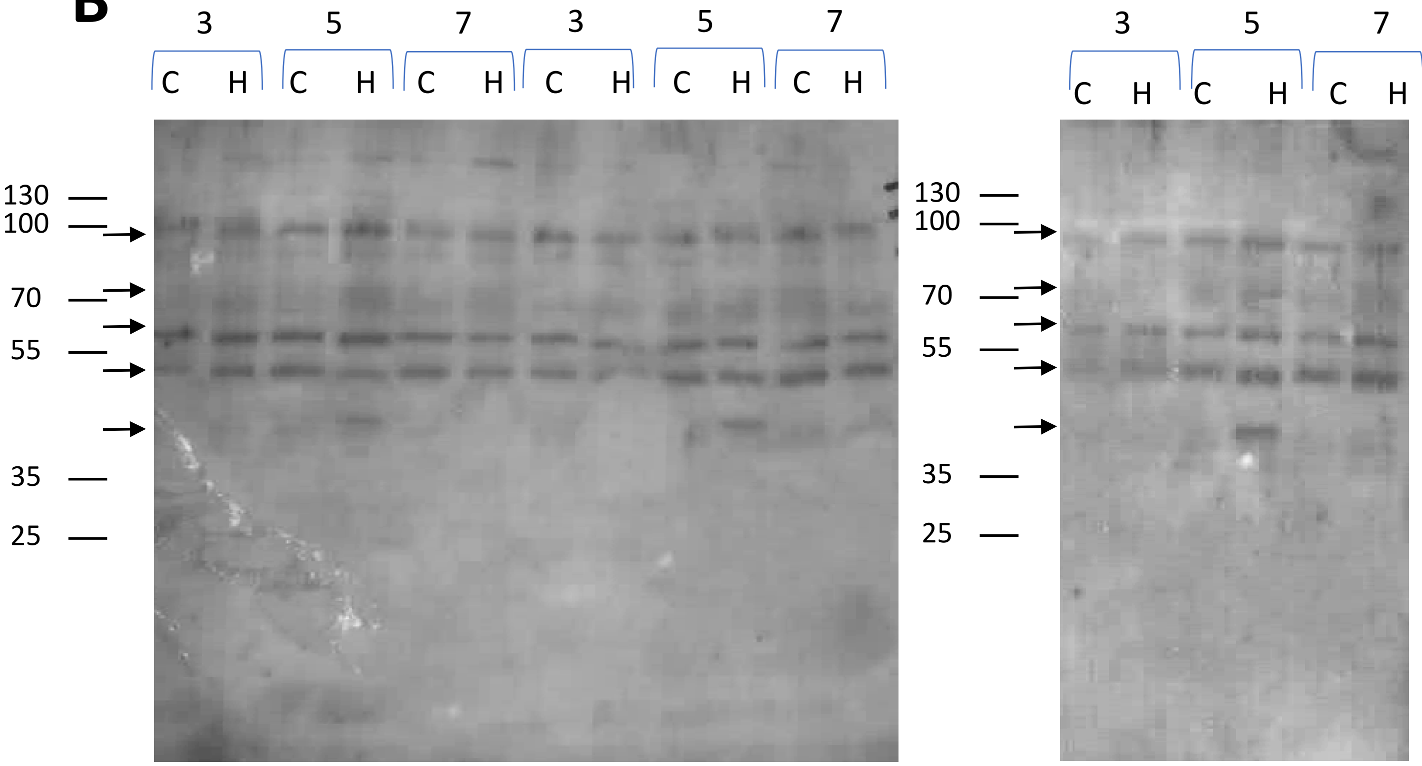

Supplementary Figure S3. Original Western blot analysis of HNE-modified proteins in kidney from females (**A**) and males (**B**) control (C) and Hfe$^{-/-}$ (H) mice at 3-, 5- and 7-months. n=3 per group. Molecular weight is indicated in KDa. Parts of blots are separately presented in Figure 3A.

Brain

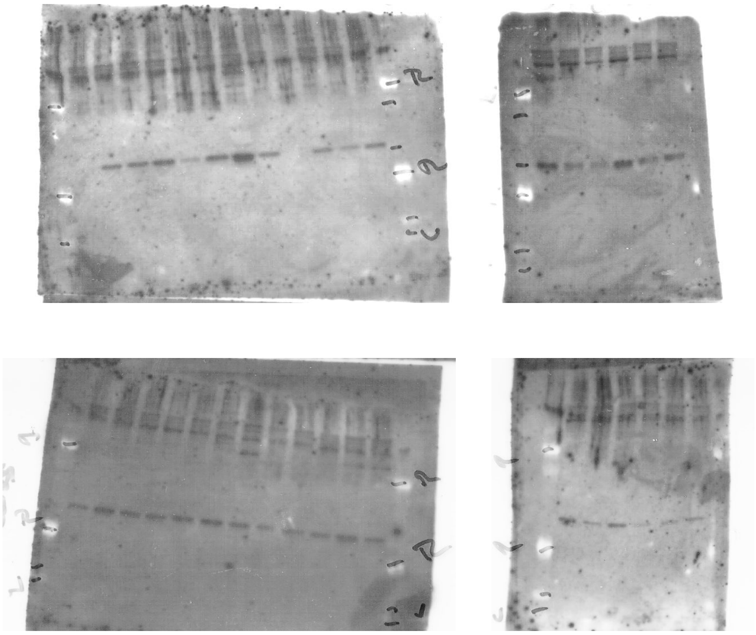

Supplementary Figure S4. Original uncropped Western blot analysis of HNE-modified proteins in brain. Blots are labelled in supplementary figure 5. Parts of blots are separately presented in Figure 3A.

Brain

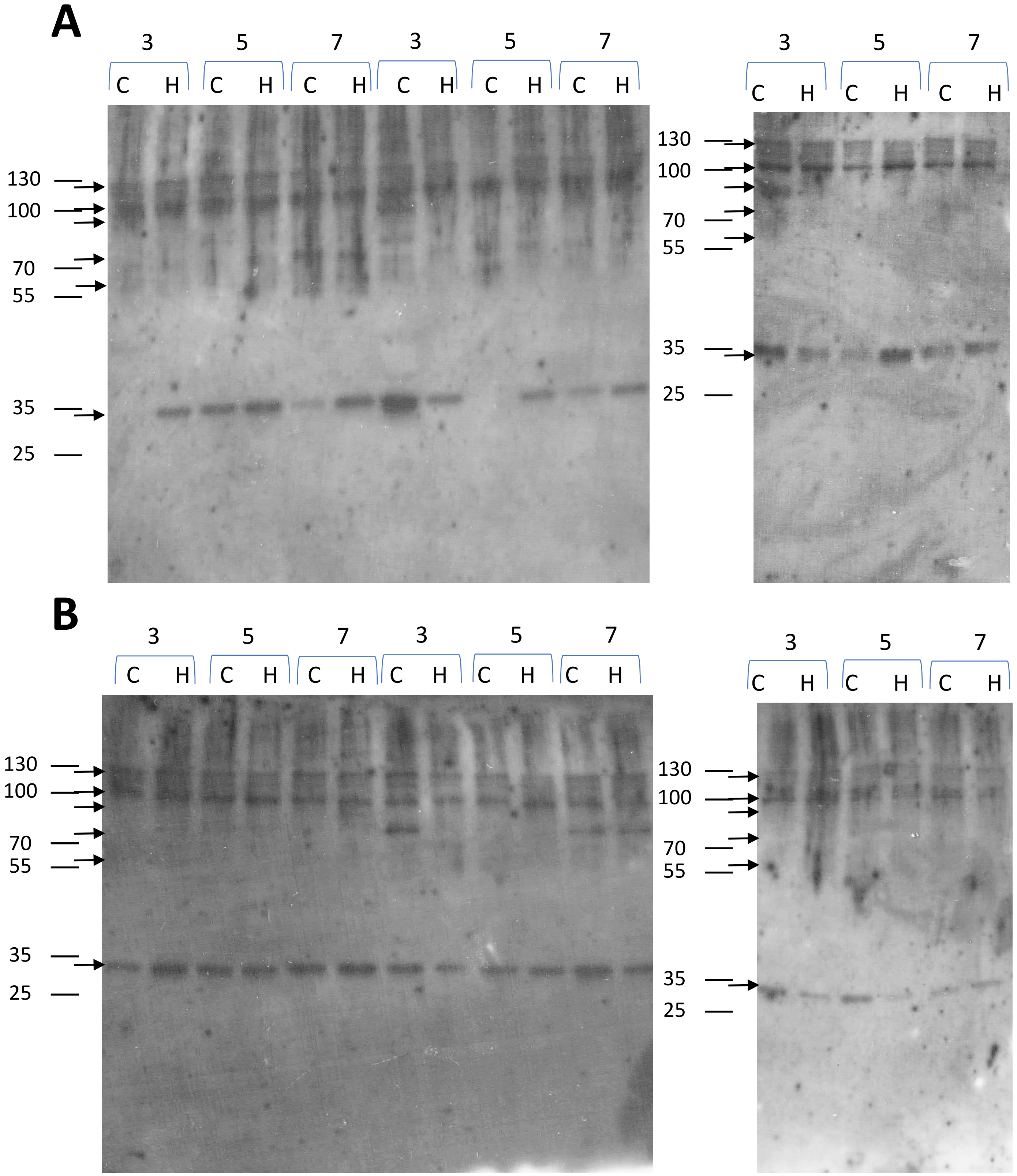

Supplementary Figure S5. Original Western blot analysis of HNE-modified proteins in brain from females (A) and males (B) control (C) and Hfe$^{-/-}$ (H) mice at 3-, 5- and 7-months. n=3 per group. Molecular weight is indicated in KDa. Parts of blots are separately presented in Figure 3A.

Heart

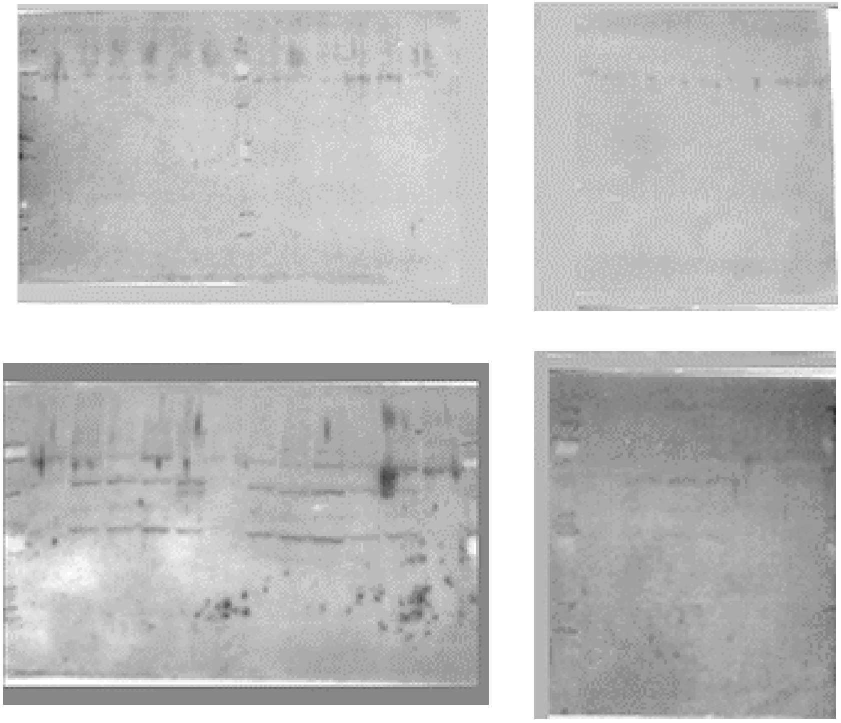

Supplementary Figure S6. Original uncropped Western blot analysis of HNE-modified proteins in heart. Blots are labelled in supplementary figure 7. Parts of blots are separately presented in Figure 3A.

Heart

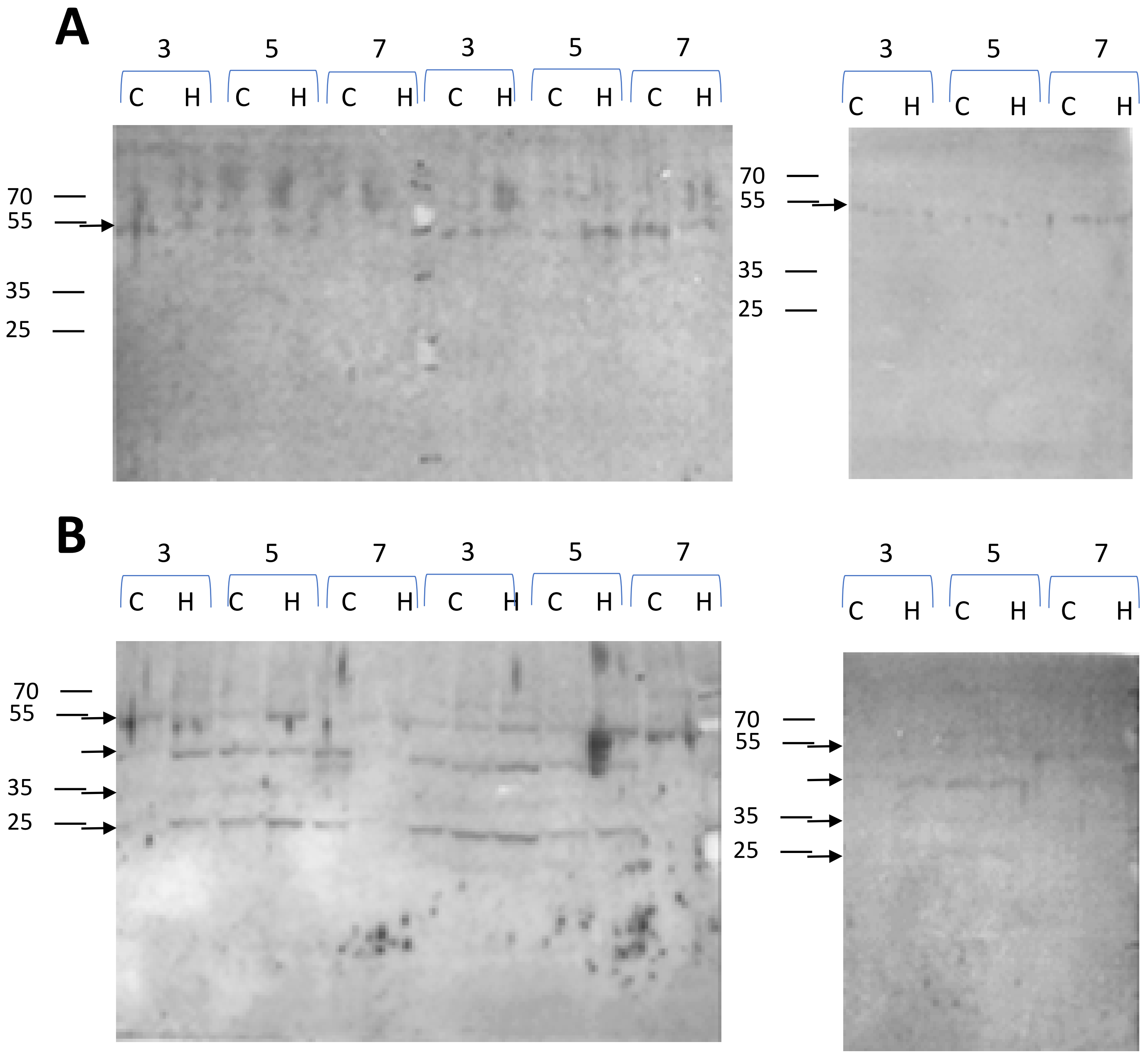

Supplementary Figure S7. Original Western blot analysis of HNE- modified proteins in heart from females (A) and males (B) control (C) and $Hfe^{-/-}$ (H) mice at 3-, 5- and 7-months. n=3 per group. Molecular weight is indicated in KDa. Parts of blots are separately presented in Figure 3A.
